# Targeted Sequencing Analysis of Predominant Histological Subtypes in Resected Stage I Invasive Lung Adenocarcinoma

**DOI:** 10.7150/jca.51405

**Published:** 2021-04-02

**Authors:** Yan Li, Yan Tan, Song Hu, Jun Xie, Zhantao Yan, Xian Zhang, Yun Zong, Han Han-Zhang, Qing Li, Chong Li

**Affiliations:** 1 Department of Respiratory Medicine, The Third Affiliated Hospital of Soochow University, 185 Juqian Road, Changzhou, China.; 2 Department of Pathology, The Third Affiliated Hospital of Soochow University, 185 Juqian Road, Changzhou, China.; 3 Burning Rock Biotech, Guangzhou, Guangdong, 510300, China.; 4 Department of Respiratory Medicine, The Affiliated Hospital of Xuzhou Medical University, 99 Huaihai Road, Xuzhou, China.

**Keywords:** adenocarcinoma, pathological subtypes, next-generation sequencing, mutational profile, immunotherapy

## Abstract

**Objective:** Lung adenocarcinoma (LADC) is classified into five main histological subtypes with distinct clinicopathologic characteristics: lepidic-predominant adenocarcinoma (LPA), acinar-predominant adenocarcinoma (APA), papillary-predominant adenocarcinoma (PPA), micropapillary-predominant adenocarcinoma (MPA) and solid-predominant adenocarcinoma (SPA). However, the mutational profiles of predominant histological subtypes have not been well defined. In this study, we aimed to reveal the genomic landscape of 5 main histological subtypes.

**Patients and Methods:** We performed next-generation sequencing (NGS) in a cohort of 86 stage I invasive adenocarcinoma (IAC) patients, using a customized panel including 168 cancer-associated genes.

**Results:** Our analysis identified a total of 302 genomic alterations. Five subtypes showed different mutation profiles with LPA, APA, PPA, MPA and SPA had an average mutation rate of 1.95 (range: 0-5), 2.56 (range: 1-6), 3.5 (range: 1-7), 3.75 (range: 1-8) and 6.05 (range: 2-12), respectively (p=4.17e-06).

Driver mutations occurred in 96.55% (83/86) of all patients. EGFR (73.3%), KRAS (9.3%), ALK (4.7%) and MET (4.7%) are the most commonly mutated lung cancer driver genes, TP53 is the top mutated tumor suppressor gene. SPA patients harbored more driver mutations and higher frequency of TP53 than LPA patients. Interestingly, *LRP1B* mutations, which has been reported to be associated with high tumor mutation burden and better response to immunotherapy, were only detected from 5 SPA patients (p=0.001). No patients from other four cohorts harbored *LRP1B* mutations.

**Conclusions:** We revealed distinctive mutation landscape of the 5 major histological subtypes of LADC, evident by distinctive average mutation rate with SPA and LPA having the highest and lowest average mutation rate, respectively. SPA patients showed higher mutation rate of LRP1B and higher rates for PD-L1 positivity, indicating that SPA patients may have better response to immunotherapy.

## Introduction

LADC is a heterogeneous tumor, accounting for almost half of all lung cancers. In 2011, the International Association for the Study of Lung Cancer, the American Thoracic Society, and the European Respiratory Society (IASLC/ATS/ERS) proposed a new histologic subtyping system for LADC. IAC was further classified into five major histologic subtypes: LPA, APA, PPA, MPA and SPA 1. The recently updated 2015 World Health Organization (WHO) classification of lung tumor is generally consistent with the 2011 IALSC/ATS/ERS classification 2. Studies have well demonstrated that there is an association between IAC subtypes and survival 3-5. Lepidic-predominant subtype was significantly related to the absence of lymph node metastasis 6 and associated with the most favorable prognostic outcome. Micropapillary and solid predominant subtypes were predictors of increased frequency of lymph node metastasis 7-10 and significantly related to disease recurrence and death 11-17. Acinar and papillary predominant subtypes had an intermediate prognosis.

Histological subtypes correlate with molecular changes. Numerous studies have evaluated the association between EGFR/KRAS/ALK mutation status and histological subtype. EGFR mutations more commonly occurred in the papillary and micropapillary subtype 18-20, and less commonly occurred in the solid subtype 21. KRAS mutations and ALK rearrangements were associated with the solid predominant subtype 21, 22.

However, previous studies have focused on several common driver genes, the mutational profiles of predominant histological subtypes have not been well defined. In this study, we aimed to reveal the genomic landscape of the 5 main histological subtypes by targeting 168 cancer-related genes.

## Methods

### Patients and sample collection

86 patients diagnosed with pathologic stage I (T1-2aN0M0) invasive lung adenocarcinoma were enrolled between January 2015 and December 2018. No patients had received any preoperative chemotherapy or radiotherapy. Tumor staging was performed according to the 8th edition of the TNM classification of the international association for the study of lung cancer (IASLC).

For each patient, surgically resected tumor was obtained immediately post-surgery. Hematoxylin-eosin (H&E) staining was performed on the tissue obtained. Two pathologists estimated and marked the predominant pattern for each tissue sample, according to the 2015 WHO criteria. The predominant pattern was defined as the pattern that occupied most of the tumor, because of small sample size, MPA was defined as an adenocarcinoma with micropapillary component exceeded 10% of the entire tumor area. The number of each subtypes were as follows: LPA (n=20), APA (n=18), PPA (n=16), SPA (n=16) and MPA (n=16). This study was approved by the Review Broad of the Third Affiliated Hospital of Soochow University.

### PD-L1 Staining

PD-L1 expression of tumor cells of each sample were determined by IHC using the PD-L1 specific DAKO 22C3 antibody, and was evaluated by a tumor proportion score (TPS). The specimens were considered PD-L1+ (TPS ≥1%) and high PD-L1+ (TPS ≥50%).

### Tissue DNA isolation and capture-based targeted DNA sequencing

Tissue DNA was extracted from the predominant pattern marked in each tissue sample using QIAamp DNA FFPE tissue kit (Qiagen) following manufacturer's instructions. A minimum of 50 ng of DNA is required for NGS library construction. Tissue DNA was sheared using Covaris M220 (Covaris, MA, USA), followed by end repair, phosphorylation and adaptor ligation. Fragments between 200-400bp from the sheared tissue DNA were purified (Agencourt AMPure XP Kit, Beckman Coulter, CA, USA), followed by hybridization with capture probes baits, hybrid selection with magnetic beads and PCR amplification. The quality and the size of the fragments were assessed using Qubit 2.0 Fluorimeter with the dsDNA high-sensitivity assay kit (Life Technologies, Carlsbad, CA). Indexed samples were sequenced on Nextseq500 (Illumina, Inc., USA) with paired-end reads and average sequencing depth of 1,000X for tissue samples. A panel of 168 genes including 68 lung cancer-related genes and 100 other genes related to cancer development, spanning 0.273 megabases (Mb) of the human genome, were used for targeted sequencing (Lung Plasma, Burning Rock Biotech, Guangzhou, China).

### Sequence data analysis

Sequence data were mapped to the reference human genome (hg19) using Burrows-Wheeler Aligner v.0.7.10 23. Local alignment optimization, duplication marking and variant calling were performed using Genome Analysis Tool Kit v.3.2 24, and VarScan v.2.4.3 25. Variants were filtered using the VarScan fpfilter pipeline, loci with depth less than 100 were filtered out. Base-calling in tissue samples required at least 8 supporting reads for single nucleotide variations (SNV) and 5 supporting reads for insertion-deletion variations (INDEL). Variants with population frequency over 0.1% in the ExAC, 1000 Genomes, dbSNP or ESP6500SI-V2 databases were grouped as single nucleotide polymorphisms (SNP) and excluded from further analysis. Remaining variants were annotated with ANNOVAR (2016-02-01 release) 26 and SnpEff v.3.6 27. Analysis of DNA translocation was performed using Factera v.1.4.3 28. Copy number variations (CNV) were analyzed based on the depth of coverage data of capture intervals. Coverage data were corrected against sequencing bias resulting from GC content and probe design. The average coverage of all captured regions was used to normalize the coverage of different samples to comparable scales. Copy number was calculated based on the ratio between the depth of coverage in tumor samples and average coverage of an adequate number (n>50) of samples without CNV as references per capture interval. CNV is called if the coverage data of the gene region was quantitatively and statistically significant from its reference control. The limit of detection for CNVs is 1.5 and 2.64 for deletions and amplifications, respectively.

### Survival analysis

The Kaplan-Meier method was used for survival rate estimation. The log-rank test was used for the comparison of survival curves between three groups. All statistical analysis was performed using R. All tests were two-sided and had a significance level of 0.05.

## Results

### Characteristics of IAC patients

Clinical characteristics of 86 IAC patients are summarized in Table 1. The median age of all patients was 64 years with a range of 36-78 years. There was no significant difference in age (P=0.511), smoking status (P=0.061) and vascular invasion (P=0.402) between the five groups. SPA group had more male (P=0.031) and stage Ⅰ B patients (P=0.014).

### PD-L1 expression status

The positive expression rate of PD-L1 was 5% (1/20), 11.1% (2/18), 43.8% (7/16), 25% (4/16), 62.5% (10/16) in LPA, APA, PPA, MPA and SPA, respectively (P=0.002). In addition, 5.6% (1/18) of APA, 12.5% (2/16) PPA, 6.3% (1/16) of MPA and 18.8% (3/16) of SPA had high PD-L1 TPS. None of LPA had high PD-L1 TPS (P<0.0001) (Table 1).

### Survival analysis

**Figure 1:**
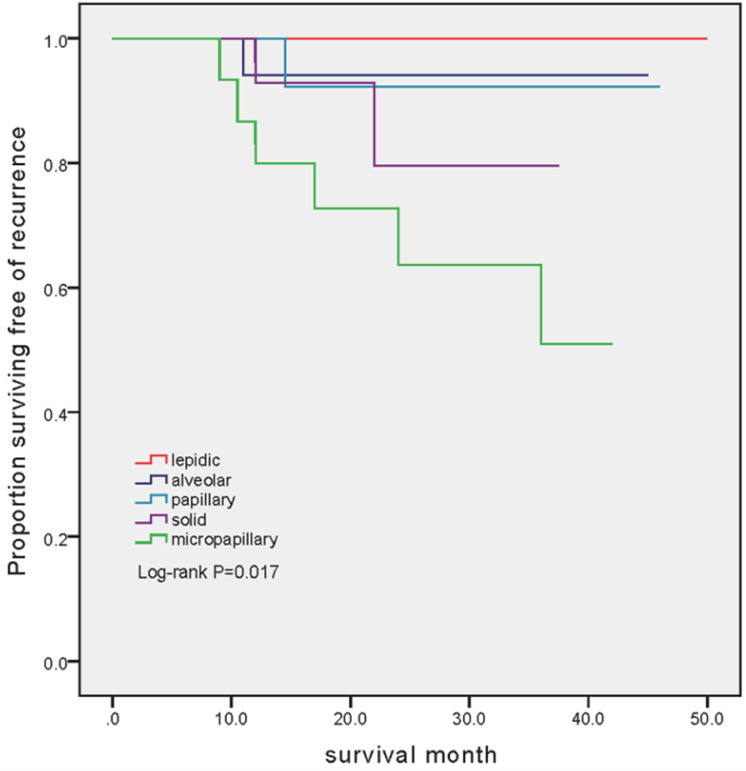
Kaplan-Meier estimates of recurrence-free survival in patients with different subtype. In the LPA group, no patient has developed recurrence (mean follow-up 29.2 months, range 9-50 months). In the APA group, 1 out of 18 (5.56%) experienced recurrent disease (mean follow-up 24.1 months, range 8.5-45 months). In the PPA group, 1 out of 16 (6.25%) experienced recurrent disease (mean follow-up 24.5 months, range 12.5-46 months). In the SPA group, 2 out of 16 (12.5%) experienced recurrent disease (mean follow-up 19.9 months, range 8.5-37.5 months). In the MPA group, 6 out of 16 (37.5%) experienced recurrent disease (mean follow-up 24.3 months, range 8.5-42 months).

The mean follow-up was 26.3 months (ranged from 8.5 to 50 months). Among 86 patients, 10 (11.6%) recurrences were identified. Disease progressions were markedly different between cohorts (P=0.017). The number of patients developed recurrent disease was 6 (37.5%), 2 (12.5%), 1 (5.56%) and 1 (6.25%) in MPA, SPA, APA and PAA group, respectively. Notably, there was no patient experienced recurrent disease in LPA group (Figure 1).

### Distinctive genomic profiles of five histological subtypes

**Figure 2:**
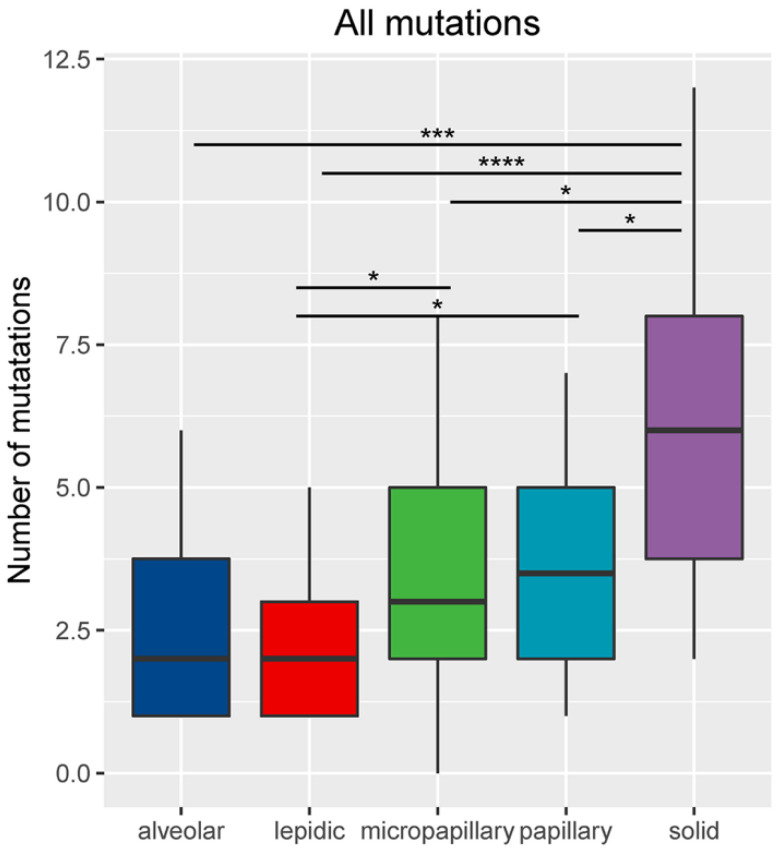
Number of mutations (including small nucleotide variations, indels, copy number variations and fusions) based on histological subtype. *Represents p<0.05, **represents P<0.01, ***represents P<0.001, ****represents P<0.0001.

For the 86 samples, we identified a total of 302 genomic alterations using a panel consisting of 168 cancer-related genes, including 161 missenses (53%), 50 copy number variances (17%), 33 insert-indels (11%), 19 nonsense (6%), 18 frameshifts (6%), 13 splicing site mutations (4%), 8 fusions (3%). One LPA patient had no detected mutation. Our data revealed that the average mutation rate was 1.95 (range: 0-5), 2.56 (range: 1-6), 3.5 (range: 1-7), 3.75 (range: 1-8) and 6.05 (range: 2-12) in LPA, APA, PPA, MPA and SPA, respectively. The number of total mutations was significantly higher in SPA than in MPA (P <0.05), PPA (P <0.05), APA (P <0.001) and LPA (P<0.0001) (Figure 2). Mutations in *EGFR*, *TP53* and *APC* were identified in all five histological subtypes. Each subtype also has subtype-specific mutations; LPA, APA, PPA, MPA and SPA have 3, 1, 8, 10 and 31 subtype-specific mutations, respectively. Table 2 shows the specific mutated genes in different groups.

### Classic lung cancer driver genes in histological subtypes of IAC

**Figure 3:**
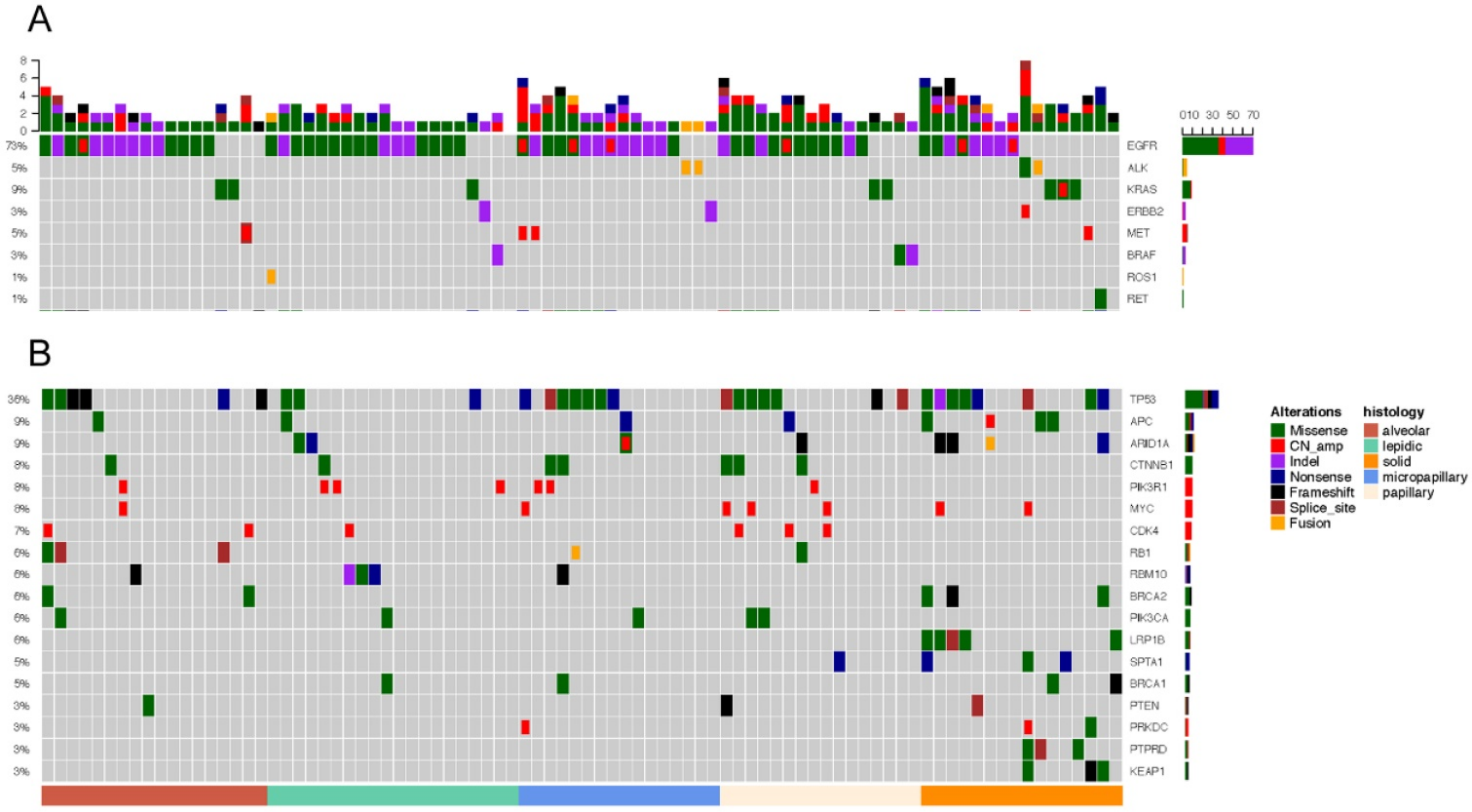
Mutation landscape of five subtypes. Each column represents a sample. The top bar plot shows the mutation number of each sample. Genetic alterations are presented by various colors. The right column indicated mutated genes. The left column indicated the percent of samples harboring gene variants.

**Figure 4:**
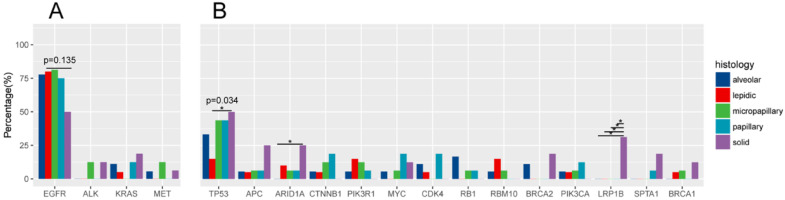
Comparison of gene mutation frequencies in five subtypes.

Driver gene mutations was identified in 96.55% (83/86) of all samples (Figure 3A). EGFR was the most frequently mutated common driver (63/86, 73.3%), followed by KRAS (8/86, 9.3%), ALK (4/86, 4.7%), MET (4/86, 4.7%), ERBB2 (3/86, 3.5%), BRAF (3/86, 3.5%), ROS1 (1/86, 1.2%), RET (1/86, 1.2%). EGFR mutations showed a trend of higher prevalence in MPA compared with SPA (13/16, 8/16), but this was not statistically significant (P=0.135) (Figure 4A).

In 20 patients with LPA, EGFR was most frequently mutated (16/20, 80%), followed by KRAS (1/20, 5%), ERBB2 (1/20, 5%), BRAF (1/20, 5%). ALK, MET, RET mutations were not detected. In 18 APA samples, mutant driver genes were EGFR (14/18, 77.8%), KRAS (2/18, 11.1%), MET (1/18, 5.6%). No ALK, ERBB2, BRAF, ROS1 and RET alterations were detected. One patient had no driver gene mutation. Among the 16 PPA patients, driver mutations were only detected in EGFR (12/16, 75%), KRAS (2/16, 12.5%), BRAF (2/16, 12.5%). PPA patients did not have mutations in ALK, ERBB2, BRAF, MET, ROS1 and RET. Among the 16 patients with MPA, driver gene mutations were detected in EGRF (13/16, 81.3%), ALK (2/16, 12.5%), ERBB2 (1/16, 6.3%), MET (2/16, 12.5%). No mutations in KRAS, BRAF, ROS, RET were detected. In 16 patients with SPA, the frequency of mutations - EGFR, KRAS, ALK, ERBB2, MET, RET - was 8/16 (50%), 3/16 (18.9%), 2/16 (12.5%), 1/16 (6.3%), 1/16 (6.3%), 1/16 (6.3%). One ALK missense patient also had concomitant ERBB2 amp. One patient was negative for driver mutations.

### TP53 and other mutations

TP53 is the most frequently mutated tumor suppressor gene (Figure.3B), with a frequency of 36.0% (31/86) of all samples and 15% (3/20) of LPA, 33.3% (6/18) of APA, 43.8% (7/16) of PPA, 43.8% (7/16) of MPA, 50% (8/16) of SPA. More TP53-mutant patients were observed in SPA than in LPA. (p=0.034) (Figure 4B). The frequencies of ARIDIA and LRP1B mutation were significantly higher in SPA than in other groups (P<0.05). Interestingly, *LRP1B* mutations, which has been reported to be associated with high tumor mutation burden and better response to immunotherapy, were only detected from 5 SPA patients (p=0.001).

## Discussion

The invasive adenocarcinoma is heterogeneous; however, the molecular features of predominant subtypes are elusive. Numerous literatures have identified the significant associations of driver gene mutations and histological subtypes of invasive adenocarcinoma. The EGFR mutation frequency was found to be higher in micropapillary, papillary, acinar and lepidic predominant component, lower in the solid predominant subtype 19, 20, 22, 29, 30. Micropapillary component can be used as a predictor of EGFR mutation 19. Micropapillary component-positive patients with EGFR mutations can benefit from EGFR-TKIs 31, 32; while solid predominant subtype is a negative response predictor for EGFR-TKI 33. ALK rearrangements were significantly associated with solid predominant subtype and component of signet-ring cells 34-38. KRAS mutations were more commonly occurred in invasive mucinous adenocarcinoma 21 and solid predominant tumors 22, 39. ROS1 fusion were closely associated with solid and acinar patterns 40.

In our study, distinct driver gene mutations were detected in different subtypes. EGFR was the most frequently shared mutated driver gene 73.3% (63/86), which is higher than 40%-50% of whole LADC population. Consistent with previous reports, we found that EGFR mutations were more frequent in MPA, less common in SPA. ALK mutations were only detected in MPA and SPA. In general, EGFR was an important factor in the beginning of lung cancer and then play a decreased role in developed lung cancer and ALK fusions may occur at a later stage in the progression of lung cancer. In addition, we observed that from LPA to SPA, the frequency of TP53 mutations significantly increased. Previous work revealed that TP53 was relatively later molecular event as a key mediator in the invasiveness of lung cancer 41, 42.

Z-Y. Dong et al. found that SPA subtype harbored a notable increase of nonsynonymous mutation and higher rate of transversion/transition based on The Cancer Genome Atlas (TCGA) and Broad database 43. We also found that even in stageⅠdiseases, SPA had more complex driver gene mutations, higher total number of mutations and more private genes. Notably, LRP1B mutation frequency was 5% (5/100) in the entire cohort, 25% (5/20) were found in SPA, and none in other four subtypes. Li Ding et al. 44 found that mutations in LRP1B was negatively correlated with acinar, papillary and bronchioloalveolar carcinoma (BAC) subtypes and positively correlated with solid subtype. LRP1B mutations, as an important cancer suppressor gene 45, are correlated with higher-TMB and neoantigen burden. Meanwhile, mutation in LRP1B were identified to be associated with better immunotherapy survival outcome in non-small cell lung cancer (NSCLC) and melanoma patients. In LRP1B mutant samples, tumor-infiltrating immune cells were more abundant, which indicated a preferable immune response status 46-48. Furthermore, PD-L1, as an immunotherapy biomarker, was significantly correlated with higher histologic grades (micropapillary and solid subtypes), compared to mediate histologic grades (acinar and papillary subtypes) and low grades (lepidic subtypes) 49-52. In our study, the incidences of PD-L1+ and high PD-L1+ lesions were significantly higher in solid subtypes. These findings suggest that SPA patients - especially those harboring *LRP1B* mutations, may benefit from immunotherapy.

Even with the same TNM stage, our study revealed the distinct mutational profile in different histological subtypes. The intrinsic mutation may determine the malignant grade of various lung adenocarcinoma subtypes. It is estimated that over 50% of patients with early-stage NSCLC will suffered recurrence after surgery 53. Among patients with lung adenocarcinoma, outcomes after surgical resection vary according to predominant histologic subtype. Many studies have reported that the presence of micropapillary and solid subtypes (predominant or even minor component) has significant prognostic value. Micropapillary and solid subtypes were associated with worse disease-free survival (DFS) and overall survival (OS) 3-5, 11, 54, higher possibility of lymph node metastasis 7-10, 55 and recurrence 13, 15, 31, 56. The recurrence hazard increased as a function of the percentage of micropapillary and solid pattern 56, while higher percentage of lepidic component was associated with a lower risk for recurrence 57, 58. Among patients who recurred, solid predominant tumors had earlier, more extra-thoracic, more multisite recurrences and worse postrecurrence survival (PRS) than those with non-solid tumors 12. In our study, SPA and MPA have higher risk of recurrence compared to those with PPA, APA and LPA (P=0.017).

Recently, the architectural classification of IAC with surgical resection has got more and more attention in clinical practice. Ming-Sound Tsao etc. 59, 60 analysed 575 patients with completely resected lung adenocarcinoma from the LACE-Bio study, and showed the first evidence that micropapillary/solid-predominant histology predict survival benefit from adjuvant chemotherapy in patients with early-stage disease. Shinsuke Sasada 61 revealed that postoperative adjuvant chemotherapy could be considered for non-lepidic predominant tumors even at stage IA. Clinically, stage I IAC patients, which are often underwent uniform treatment and follow-up, might need distinctive therapeutic care based on the histological subtypes with divergent molecular basis.

Our study has several limitations. Firstly, tumor subtypes were extracted from different patients, as they didn't share identical genetic background and relative exposure history, we were unable to detect evolutionary trajectories of the five subtypes. Secondly, the median follow-up time was relatively short, we can't fully investigate the relationship between histologic subtypes, molecular subtypes and clinical efficacy of different treatment protocols. Thirdly, due to the small size of sample with micropapillary occupied most of the area, we selected tumors with micropapillary component exceeded 10% of the entire tumor area as MPA. The genetic mutation profile of MPA may be affected by sample selection.

## Conclusions

In summary, in resected stage I IAC, prognoses of solid or micropapillary predominant subtypes were apparently worse than that of other subtypes, different histological subtypes had distinct mutational profiles. SPA harbored more complex mutation profile even at stage I and higher mutation rate of LRP1B. The rates for PD-L1 positivity and high TPS were significantly higher in SPA. Clinically, SPA patients may benefit from immunotherapy. Stage I IAC patients, which are often underwent undifferentiated treatment and follow-up, might need distinctive therapeutic care based on the histological subtypes with unique genetic profiles.

## Figures and Tables

**Table 1:** Patient clinical characteristics

	n=86	n=20	n=18	n=16	n=16	n=16	
**Age, years**							
Range	36-78	40-74	48-74	45-72	43-78	36-75	0.511
Median	64	59	64.5	64	65	64	
**Gender**							
Male	46	8	8	7	12	11	0.031
Female	40	12	10	9	4	5	
**Smoking status**							
Smoker	23	2	3	6	4	8	0.061
Never-smoker	63	18	15	10	12	8	
**Clinical stage**							
Stage IA	67	20	13	12	9	13	0.014
Stage IB	19	0	5	4	7	3	
**Vascular invasion**							
Presence	2	0	0	1	0	1	0.402
Absence	84	20	18	15	16	15	
**PD-L1+**							
≥1%	24	1	3	6	10	4	0.002
<1%	62	19	15	10	6	12	
**High PD-L1+**							
≥50%	7	0	1	2	3	1	<0.0001
<50%	79	20	17	14	13	15	

**Table 2:** Mutated genes private in LPA, APA, PPA, MPA and SPA group

Genes private in LPA
MTOR ROS1 JAK1
Genes private in APA
INHBA
Genes private in PPA
KIT PDGFRA MAP3K13 CHEK2 U2AF1 EPHB1 TGFBR2 FGFR2
Genes private in MPA
IL7R NTRK1 SETD2 PALB2 VEGFA MEN1 FBXW7 DNMT3A ATR PMS2
Genes private in SPA
EPHA5 ERBB4 CDKN1B EPHA7 HIST1H3B NOTCH1 CD274 PAK5 LRP1B MLH1 TERT KDR SOX9 PPP2R1A RET EPHA3 RUNX1 STK11 PIK3CG RAD51C FAT3 MSH2 GRIN2A FGFR1 IKZF1 NTRK3 KEAP1 PTPRD KDM5A ATM ESR1

## Abbreviations

LADC: lung adenocarcinoma
LPA: lepidic-predominant adenocarcinoma
APA: acinar-predominant adenocarcinoma
PPA: papillary-predominant adenocarcinoma
MPA: micropapillary-predominant adenocarcinoma
SPA: solid-predominant adenocarcinoma
NGS: next-generation sequencing
IAC: Invasive adenocarcinoma
IASLC/ATS/ERS: International Association for the Study of Lung Cancer, the American Thoracic Society, and the European Respiratory Society
WHO: World Health Organization
SNV: single nucleotide variations
INDEL: insertion-deletion variations
SNP: single nucleotide polymorphisms
CNV: copy number variations
TCGA: The Cancer Genome Atlas
BAC: bronchioloalveolar carcinoma
NSCLC: non-small cell lung cancer
DFS: disease-free survival
OS: overall survival
PRS: postrecurrence survival
